# Predicting the Detectability of Thin Gaseous Plumes in Hyperspectral Images Using Basis Vectors

**DOI:** 10.3390/s100908652

**Published:** 2010-09-17

**Authors:** Kevin K. Anderson, Mark F. Tardiff, Lawrence K. Chilton

**Affiliations:** Pacific Northwest National Laboratory, PO Box 999, Richland, WA 99352, USA; E-Mails: kevin.anderson@pnl.gov (K.K.A.); mark.tardiff@pnl.gov (M.F.T.)

**Keywords:** plume, detection, LWIR, basis vectors, NECL

## Abstract

This paper describes a new method for predicting the detectability of thin gaseous plumes in hyperspectral images. The novelty of this method is the use of basis vectors for each of the spectral channels of a collection instrument to calculate noise-equivalent concentration-pathlengths instead of matching scene pixels to absorbance spectra of gases in a library. This method provides insight into regions of the spectrum where gas detection will be relatively easier or harder, as influenced by ground emissivity, temperature contrast, and the atmosphere. Our results show that data collection planning could be influenced by information about when potential plumes are likely to be over background segments that are most conducive to detection.

## 1. Introduction

The value of hyperspectral imagery in detecting evidence of thin gaseous plumes is dependent upon the ability of the analysis tools to detect those materials when they are present. If an image collection mission is being planned, information should be available regarding the scene background and the anticipated materials of interest. In this paper we investigate methods for using image analysis tools to predict the minimum detectable concentration-pathlength (MDCL) for plumes over specific backgrounds prior to image collection. The intent is to develop an approach for determining under what conditions gases of interest can be detected over specific backgrounds and at what minimum concentration-pathlengths.

Estimating MDCLs for thin gaseous plumes using thermal imaging data is complicated by many factors. Methods for gas plume detection have been studied extensively and are reviewed by various authors [1–4]. Very often the approach is to evaluate specific gases over specific backgrounds and temperature emissivity (TE) contrasts. The difficulties with this approach for mission planning is that small gas libraries result in efficient searching but risk missed detections because member gases may not cover all the gases in the image. Large libraries result in slower searching and can have multiple detections because of spectral feature overlap.

An alternative approach to the detection problem with gas libraries is described by Chilton and Walsh [5]. They use a set of basis vectors (BV) consisting of one BV for each spectral channel. The BV for channel *n* has a 1 in the *n*-th location and zeros elsewhere. Their results show that applying a whitened-matched filter to each BV in succession will identify spectral channels with anomalous activity. The library in this case is the set of BVs that correspond to each spectral channel and is defined by the resolution and bandwidth of the image. This approach is useful for detection because it spans the full spectral dimension of the image and is agnostic to individual gas characteristics, thus resolving the issue of missed detections because of mismatches between image gases and library members.

In this paper we extend the application of BVs to estimate the noise-equivalent concentration-pathlength (NECL) for pixels in an image or image segment, relate the NECL to the signal-to-noise ratio (SNR) for an image or image segment, and estimate the MDCL for gases that have a single dominant spectral peak. We validate our MDCL results by injecting gases into an AHI image and using whitened-matched filtering to get empirical probabilities of detection (*P_d_*) and false detection probabilities (*P_fa_*). We compare the empirical results to the MDCL predictions at those *P_d_* and *P_fa_* values. Extension of these results to gases with multiple peaks warrants further research.

## 2. Method Development

In this section, we present the assumed physics-based radiance model and the NECL estimation method using unit basis vectors instead of actual gas absorbance spectra.

### 2.1. Physics-based Radiance Model

The three-layer physics-based radiance model at a pixel is the same as that considered by Chilton and Walsh [5]. For a pixel with a plume made up of *N_c_* gases, as a function of wavelength, *λ* (in *μ*m):

(1) $$L_{\mathrm{obs}}(\lambda )=\tau_{a}(\lambda )\;(B(T_{p};\lambda )-{ɛ}_{g}(\lambda )\;B(T_{g};\lambda ))\sum_{j=1}^{N_{c}}c_{j}A_{j}(\lambda )+\tau_{a}(\lambda ){ɛ}_{g}(\lambda )B(T_{g};\lambda )+L_{u}(\lambda )+n(\lambda )$$

where *L_obs_*(*λ*) represents sensor-recorded radiance in W/(*m*^2^ *sr * *μ*m) at wavelength *λ*, *τ_a_*(*λ*) and *ε_g_*(*λ*) are dimensionless terms representing the atmosphere transmissivity and ground emissivity, respectively, *B*(*T*; *λ*) has radiance units and is Planck’s Blackbody function, *T_p_* and *T_g_* (K) represent the plume and ground temperature, respectively, *A_j_*(*λ*) is the absorbance coefficient of gas *j* in (*ppm-m*)^−1^, *c_j_* is the concentration-pathlength of gas *j* in *ppm-m*, *L_u_*(*λ*) is the atmospheric upwelling radiance, and *n*(*λ*) includes unmodeled effects and sensor noise [2]. We assume that the hyperspectral image has been analyzed and reasonable estimates of ground temperature and ground emissivity have been produced at each pixel; available through the use of a tool such as Optimized Land Surface Temperature and Emissivity Retrieval (OLSTER) algorithm developed by Boonmee *et al*. [6]. Ground truth information about the background may be available for a collection target that has been monitored over a period of time.

The mean-adjusted radiance (subtracting the average of the off-plume pixels) is

(2) $$r(\lambda )=L_{\mathrm{obs}}(\lambda )-{\bar{L}}_{\mathrm{off}}(\lambda )=\tau_{a}(\lambda )C(\lambda ;T_{p},T_{g},{ɛ}_{g})\sum_{j=1}^{k}c_{j}A_{j}(\lambda )+\eta (\lambda )$$

where *C*(·) is the temperature-emissivity contrast

(3) $$C(\lambda ;T_{p},T_{g},{ɛ}_{g})=B(T_{p};\lambda )-{ɛ}_{g}(\lambda )B(T_{g};\lambda )$$

and *η*, a vector, contains clutter and noise terms and is approximately zero-mean with covariance matrix Σ.

In linear algebra terms, across the spectral channels, Equation 2 becomes the statistical regression model

(4) r=Xβ+η

where

(5) $$X=\tau_{a}⊙C⊙A$$

*τ_a_* is the atmospheric transmissivity vector, ***C*** is the temperature-emissivity contrast vector, ***A*** is a matrix whose columns are the gas absorbances, and *β* is a vector of concentration-pathlengths.

### 2.2. Noise-Equivalent Concentration-Pathlength

The noise-equivalent concentration-pathlength (NECL) is a measure of the uncertainty in the quantification of a particular gas for each pixel in hyperspectral imagery. In statistical terms, the NECL of a particular gas is the estimated standard deviation of the weighted least-squares regression estimate of the concentration-pathlength of the gas for non-plume pixels. The NECL is equivalent to the amount of gas which gives a SNR of 1 [7]. Such a quantity is often used to produce a minimal detectable concentration-pathlength, e.g., typically MDCL = 4 × NECL, where 4 is the sum of z-scores of the Gaussian distribution associated with the *P_d_* ≈ 0.95 and *P_fa_* ≈ 0.05. Empirical estimates of NECL values are typically calculated from hyperspectral imagery for each gas. We propose an approach to generalized NECL values using basis vectors.

For a gas of interest, the empirical single-gas NECL is calculated by first choosing a likely plume temperature; fitting the whitened matched filter for that gas at every off-plume pixel; and then taking the standard deviation of those matched filter outputs. That is, let ***A*** in Equation 5 be the absorbance spectrum of the gas of interest. Then for each off-plume pixel (and its ground temperature and ground emissivity) compute the matched filter output

(6) $$m=(X'\Sigma^{-1}r)/(X'\Sigma^{-1}X).$$

The NECL of that gas at the chosen plume temperature is the standard deviation of the *m* values. A further refinement is to compute the NECL for each pixel type in image segments (with the segment-specific mean-adjustment in Equation 2 and segment-specific Σ in Equation 6).

Thus, for each gas in a library of candidates, for each pixel type, and for various plume temperatures, NECL values may be estimated. The success of this method depends on having the plume chemicals in the search library. If the plume chemicals are not known, choosing the gas search library can be a challenge. Due to these factors, we propose a method independent of the chemicals in the plume, namely, using a set of surrogate spectra which span the spectral vector space.

The simplest set of basis vectors are the coordinate unit vectors. For a *N_λ_* channel hyperspectral instrument, the *N_λ_* basis vector NECL values (BV-NECL) are computed by replacing ***A*** in Equation 5 with the *N_λ_* basis vectors, one at a time. Smaller NECL values indicate lower variability or noise and an easier detection environment. With BV-NECL values, the assessment of relative ease of detection can be made on a channel-by-channel basis, indicating spectral regions where gases will be easier or harder to detect, given the ground emissivity, temperature contrast, and atmosphere. Because the basis vectors are not scaled to the appropriate units of absorbance spectra, the BV-NECL values are not in the units of *ppm-m*. The BV-NECL values may be compared in relative terms. The next section demonstrates that BV-NECL values in *ppm-m* units can be estimated for gases with a single dominant peak. Converting BV-NECL values to *ppm-m* units for multi-peak gases is an area for further investigation.

## 3. Application

As an illustration of the method proposed above, the AHI image with no plume was used. This image was collected with the Airborne Hyperspectral Imager built by the University of Hawaii and provided to us by the Chester F. Carlson Center for Imagery Science at Rochester Institute of Technology (RIT). The spectra are in 50 channels corresponding to wavelengths from 8.094 to 11.533 *μ*m. The ground temperatures and ground emissivities were extracted from the image using the OLSTER algorithm developed by Boonmee *et al*. [6] at RIT.

**Figure 1.**
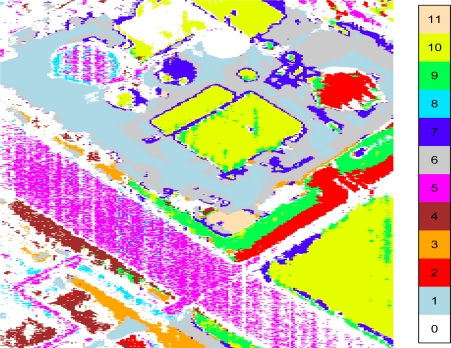
Segmented AHI Image. The 11 pixel group assignments are indicated. White areas are unassigned.

A k-th nearest neighbor approach was used to extract 11 endmembers from the ground emissivities. Each pixel was assigned to groups associated with these 11 endmembers based on the correlation of its ground emissivity to the endmembers. If no correlation was greater than 0.8, the pixel was not assigned to any of the 11 groups, which created a “group 0” of unassigned pixels. This conservative approach to image segmentation left a large proportion of the pixels unassigned. Having 11 endmembers to segment the image and using 0.8 as the minimum correlation coefficient value are arbitrary choices to get clear distinctions among the groups and lower the within-group variances to demonstrate the BV-NECL method. The numbers of pixels assigned to the 12 groups are given in Table 1. There were enough pixels in each group to calculate by-group BV-NECL values. Figure 1 shows the segmentation of the AHI scene into the 11 groups.

**Figure 2.**
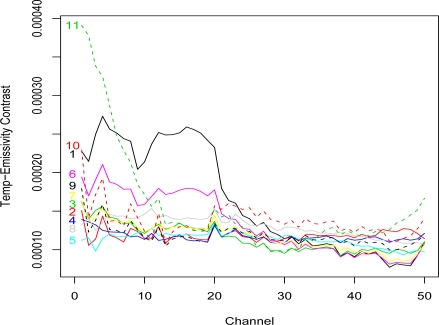
Endmember Temperature-Emissivity Contrasts. A plume temperature 5 °K hotter than the average ground temperature of each pixel group was used to compute the temperature-emissivity contrasts for the endmembers.

The robust averages and standard deviations of the ground temperature of the pixels in each group are also given in Table 1. For comparison, the robust average and standard deviation of the ground temperature for the unsegmented scene are 308.7 °K and 5.87 °K, respectively. These individual group average ground temperatures and a 5 °K hotter plume temperature were used to compute the temperature-emissivity contrast for each of the 11 endmembers, see Figure 2.

The robust variancemethod used here was developed by Cressie and Hawkins [8]. The robust variance formula for a random sample of size *n*, {*X*_1_, *X*_2_, . . ., *X_n_*}, is

(7) $$V={\left(\frac{1}{n}\sum_{i=1}^{n}{\left|X_{i}-\bar{X}\right|}^{1/2}\right)}^{4}/(0.457+0.494/n),$$

where *X̄* is either the mean or trimmed-mean of the sample. We used the middle 90% of the data to calculate a 5% trimmed mean.

The pixels in each of the 11 groups were mean-centered and then whitened match filtered using the set of basis vectors instead of a particular gas and 7 different plume temperatures: −5, 0, +2, +5, +10, +15, and +20 °K from the average ground temperature of the group. The BV-NECL values for each group were then estimated using a robust standard deviation of the whitened match filter results. The trimmed mean and robust variance method were used to estimate the BV-NECL values in order to stabilize the estimates in the presence of extreme values.

**Figure 3.**
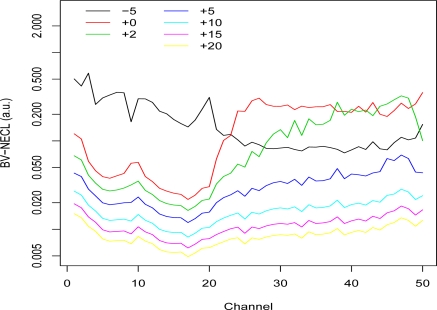
Segmented Basis Vector NECL Values: Group 1. NECL values for the 50 unit vectors (corresponding to the 50 spectral channels) were computed at 7 different plume temperatures from −5 °K to +20 °K more than the average ground temperature 311.6 °K. Only the pixels assigned to Group 1 were used in the calculations.

**Figure 4.**
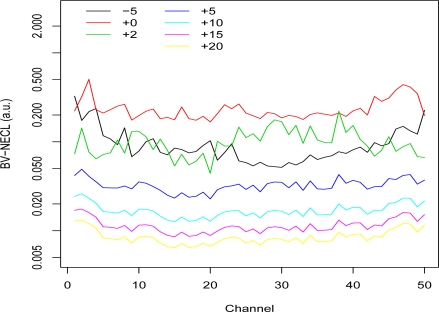
Segmented Basis Vector NECL Values: Group 3. NECL values for the 50 unit vectors (corresponding to the 50 spectral channels) were computed at 7 different plume temperatures from −5 °K to +20 °K more than the average ground temperature 311.0 °K. Only the pixels assigned to Group 3 were used in the calculations.

**Figure 5.**
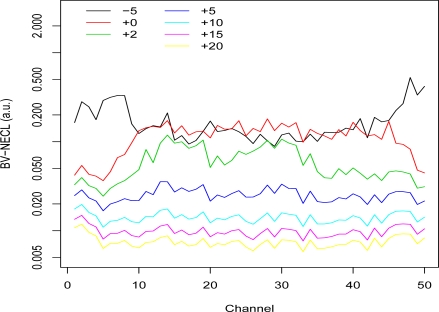
Segmented Basis Vector NECL Values: Group 11. NECL values for the 50 unit vectors (corresponding to the 50 spectral channels) were computed at 7 different plume temperatures from −5 °K to +20 °K more than the average ground temperature 311.9 °K. Only the pixels assigned to Group 11 were used in the calculations.

Figures 3, 4, and 5 show plots of the BV-NECL values for three of the pixel groups. Groups 1 and 11 were chosen because their temperature-emissivity contrasts were most different from the others and Group 3 was chosen to represent the other groups. Two general phenomena are demonstrated with these plots. First, as the temperature contrast between the ground and plume increases, the BV-NECL values decrease and become more similar across the spectral range of the data [2]. This is an interesting outcome when plume temperature exceeds ground temperature because, as one can see in Figure 3, changes in detectability are much larger in the harder to detect region of channels 30 through 50 than in channels 10 to 20. The implication is that the detectability of a plume in an image will be at least partly controlled by its extent and the cooling or heating it undergoes to equilibriate to ambient atmospheric temperature. A gas with spectral activity in channels 10 to 20 is likely to be easier to detect than a gas with spectral activity in channels 30 to 50.

The second interesting feature of these plots is the comparison of the −5 °K, 0 °K, and +5 °K temperature lines. The results were produced using the 1976 standard atmosphere, so the differences result from the ground emissivities for each group. Walsh *et al*. [9] present an analysis of how temperature contrast and emissivity influence detection. One can see that the channels where the differences between −5 °K and 0 °K BV-NECL values are large in Figures 3 and 5 correspond to the channels where the endmembers (1 and 11) in Figure 2 have higher temperature-emissivity contrasts. The flat temperature-emissivity contrast of endmember 3 corresponds to the flat BV-NECL values in Figure 4. The implication to mission planning is that data collection might be managed for complex scenes with multiple backgrounds to detect potential plumes over those segments of the scene most conducive to their detection.

**Figure 6.**
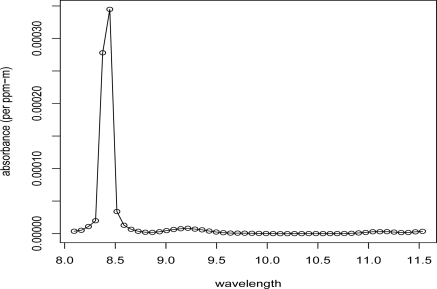
Gas 7 Absorbance Spectrum.

The BV-NECL values can be used to make inference about the detection capability of single-peak gases. The appropriate BV-NECL values can be scaled by dividing by the maximum absorbance of the gas to estimate its NECL values. For example, consider Dibromoethane (Gas 7) from the AHI chemical library shown in Figure 6. For this gas, we are concerned with the BV-NECL values for the 6th channel where the maximum absorbance is 0.000345 (*ppm-m*)^−1^. We can compare the scaled 6th channel BV-NECL values to the actual empirical NECL values computed for Gas 7 across the 11 groups (see Table 2 for comparisons using a +5 °K temperature plume). We set a detection critical value for a 1% probability of false alarm (*P_fa_*) at 2.326 ×BV-NECL/0.000345. We also estimate the minimum detectable level that gives 95% probability of detection (*P_d_*) at that 1% *P_fa_* as (2.326 + 1.645) × BV-NECL/0.000345 = 3.971 × BV-NECL/0.000345. Here 2.326 and 1.645 are z-scores of the Gaussian distribution corresponding to tail probabilities of 0.01 and 0.05.

Table 2 gives the estimated MDCL for a +5°K plume temperature across the 11 groups in the AHI image. The table also provides the empirical *P_fa_* and *P_d_* values for critical values set using the BV-NECL values. All of the empirical *P_fa_* values are higher than the nominal 1% *P_fa_*. The empirical *P_d_* values, assuming a gas concentration-pathlength at the BV-NECL-based estimated MDCLs, are only slightly lower than the nominal 95%. The differences between the nomimal and empirical *P_d_* and *P_fa_* values are due to differences in the tails of the within-pixel group whitened matched filter distributions compared to the Gaussian distribution. In this case, the tails of the empirical distribution are heavier than the Gaussian distribution. Using the Gaussian to make inference about quantiles for small probabilities (less than about 2%) are inaccurate. The heavier tails are probably due to mixed pixels.

The estimated MDCLs range from 218 to 540 *ppm-m* with three values below 280, three values between 320 and 400, three values between 400 and 500, and two values over 500. The range and distribution of these results indicate that segmenting the scene reveals important differences in detectability over the various backgrounds that warrant consideration in planning a data collection.

This study used a gas with a single dominant spectral peak and whitened matched filtering to produce the empirical detection estimates. The single-peak gas was selected to demonstrate the BV-NECL method because it represents the simplest challenge and any confounding factors thatmight be associated with multi-peak gases are eliminated. If the method failed to provide useful results with this gas, then any promising results with multi-peak gases would very likely be arbitrary. Initial efforts to apply the BV-NECL technique to multi-peak gases indicate that extending the method to these gases will require further development. Additionally, we used whitened matched filtering because it is a common technique for gas detection in hyperspectral images. How the BV-NECL technique performs with other estimators has not been investigated.

## 4. Conclusions

We presented a method for predicting the detectability of thin gaseous plumes in hyperspectral images. The novelty of this method is that using basis vectors for each of the spectral channels of a collection instrument to calculate NECL values instead of library gases provides insight into regions of the spectrum where gas detection will be relatively easier or harder, as influenced by ground emissivity, temperature contrast, and atmosphere. We also relate the three-layer physics-based radiance model to NECL values, to SNRs, and finally to MDCLs.

We segmented an AHI image and analyzed it with these techniques. Our results indicate that there are meaningful differences across the MDCLs calculated for the scene segments with a factor of 2.5 between the highest and lowest MDCLs (540 *ppm-m* versus 218 *ppm-m*). The implication is that data collection planning could be influenced by information about when potential plumes are likely to be over background segments that are most conducive to detection. Our results also show that these considerations are most important with small temperature contrasts between the ground and plume. As the difference in temperature increases, the BV-NECL values get smaller, indicating that gases are easier to detect, and channel-to-channel differences across BV-NECL values decrease.

The example we present is for a single-peak gas. Our results across the 11 scene segments for this gas and for other single-peak gases indicate that we get very good agreement (within a few percent) between the scaled BV-NECL values and empirical NECL values estimated by mean-centering and whitened match filtering each of the scene segments with the basis vectors. Estimating scaled NECL values and MDCLs for multi-peak gases is a challenging problem and an area for additional research.

## Figures and Tables

**Table 1.** Group segmentation summary.

Pixel Group	Number of Pixels	Average Ground Temperature (K)	Standard Deviation (K)
0	40,605	305.8	4.84
1	14,407	311.6	3.54
2	4,716	307.1	1.82
3	2,627	311.0	2.17
4	3,588	310.1	1.97
5	12,049	301.5	0.59
6	14,675	311.9	3.15
7	6,051	311.1	2.85
8	1,530	302.1	1.20
9	5,876	310.0	2.50
10	17,752	315.0	4.70
11	924	311.9	2.73

**Table 2.** Comparison of estimated and empirical detectability for Gas 7 using +5 °K plume temperature.

Pixel Group	Number of Pixels	Scaled BV-NECL (*ppm-m*)	Empirical NECL (*ppm-m*)	Empirical *P_fa_* (nominal 1%)	Estimated MDCL (*ppm-m*)	Empirical *P_d_* (nominal 95%)
1	14,407	55.0	56.6	2.33%	218	94.1%
2	4,716	127.7	118.7	1.42%	507	95.1%
3	2,627	87.9	85.7	2.09%	349	94.5%
4	3,588	101.8	100.1	2.90%	404	94.6%
5	12,049	99.0	86.8	2.34%	393	97.0%
6	14,675	80.6	85.2	2.64%	320	93.2%
7	6,051	123.0	124.1	2.89%	488	93.2%
8	1,530	70.0	65.2	1.63%	278	94.8%
9	5,876	136.0	131.3	2.26%	540	93.9%
10	17,752	117.5	111.9	2.32%	467	93.1%
11	924	57.8	57.1	1.52%	230	94.4%

## References

[1] Burr T, Foy B, Fry H, McVey B (2006). Characterizing Clutter in the Context of Detecting Weak Gaseous Plumes in Hyperspectral Imagery. Sensors.

[2] Burr T, Hengartner N (2006). Overview of Physical Models and Statistical Approaches forWeak Gaseous Plume Detection using Passive Infrared Hyperspectral Imagery. Sensors.

[3] Gallagher N, Sheen D, Shaver J, Wise B, Shultz J (2003). Estimation of trace vapor concentration-pathlength in plumes for remote sensing applications from hyperspectral images. Proc. SPIE.

[4] Theiler J, Foy BR, Fraser AM (2005). Characterizing non-gaussian clutter and detecting weak gaseous plumes in hytperspectal imagery. Proc. SPIE.

[5] Chilton L, Walsh S (2009). Detection of gaseous plumes using basis vectors. Sensors.

[6] Boonmee M, Schott JR, Messinger DW (2006). Land surface temperature and emissivity retrieval from thermal infrared hyperspectral imagery. Proc SPIE.

[7] Ben-David A, Ifarraguerri A, Samuels AC (2003). Correlation spectroscopy with diffractive grating synthetic spectra and orthogonal subpace projection filters. Opt. Eng.

[8] Cressie N, Hawkins DM (1980). Robust estimation of the variogram: I. Journal of the International Association for Mathematical Geology.

[9] Walsh S, Chilton L, Tardiff M, Metoyer C (2008). Effect of the temperature-emissivity contrast on the chemicl signal for gas plume detection using thermal image data. Sensors.

